# Exploring the temporal correlations of factors affecting traffic safety on mountain freeways: Through new crash frequency modelling methods

**DOI:** 10.1371/journal.pone.0319831

**Published:** 2025-04-08

**Authors:** Liang Zhang, Zhongxiang Huang, Aiwu Kuang, Jie Yu, Lei Zhu, Songtao Yang

**Affiliations:** 1 School of Traffic and Transportation Engineering, Changsha University of Science and Technology, Changsha, China; 2 Civil Engineering, Hunan City College, Yiyang, China; Huazhong University of Science and Technology, CHINA

## Abstract

The potential factors contributing to safety risks on mountainous freeways exhibit significant seasonal clustering and temporal correlations. However, these temporal characteristics have not been accurately captured by existing crash modeling methods, which severely compromise model fit and may lead to erroneous conclusions. This study makes three major contributions. Firstly, a multidimensional crash dataset involving design features, traffic conditions, pavement performance, and weather conditions was established based on eight quarterly datasets of mountain freeways in China. Secondly, two new crash modeling methods considering temporal correlations were proposed. The first model embedded an autoregressive structure and a time linear trend function within a Poisson model, while the second model incorporated an autoregressive structure and time-varying regression coefficients within a Poisson model. The superiority of the new models over seven existing time-correlated models was validated in terms of goodness-of-fit and prediction accuracy, and the significant associations between crash frequencies across different quarters were also confirmed. Moreover, this study quantitatively analyzed the causes of crash frequency on mountainous freeways in China, revealing several significant conclusions. For instance, special road sections such as interchanges, tunnels, and service areas exhibit higher crash risks. Increased traffic volumes, especially with a higher proportion of trucks, are associated with elevated crash risks. Enhancing pavement smoothness and skid resistance was found to effectively mitigate crashes. Moderate rainfall increases crash risks, whereas heavy rainfall alters travel plans and paradoxically reduces crash frequency. To the best of our knowledge, this study introduced the first temporal correlation modeling method specifically addressing the unique temporal characteristics of safety-influencing factors on China’s mountainous freeways, offering valuable insights for the development of effective safety countermeasures.

## 1. Introduction

The complex geometric conditions of mountain freeways—such as sharp curves, long steep slopes, and frequent environmental changes (including numerous tunnels and bridges)—result in a significantly higher crash rate compared to other types of freeways [1]. According to data from China’s traffic safety authorities [2, 3], in 2023, the crash rate, fatality rate, and injury rate per 100 kilometers on mountain freeways in China were 3.0 times, 5.1 times, and 3.8 times higher, respectively, than those on regular freeways, which underscored the persistent (and even rising) risks to life and property faced by users of China’s mountain freeways. Therefore, it remains crucial to develop precise crash models (safety performance functions) that thoroughly understand the mechanisms of freeway crashes which can facilitate the design of appropriate geometric strategies and behavioral interventions, aiming to reduce casualties and operational economic losses on mountain freeways.

In safety research, crash frequency models were widely used for causal analysis of safety and identification of crash black spots [4–6]. It is worth noting that different crash modelling approaches focus on different directions of improvement, depending on the properties of the crash scenario data. [7–11]. For example, random parameter Poisson/negative binomial models address unobserved heterogeneity [12, 13]., Poisson-lognormal models handle low sample means or small sample sizes [14–15], and conditional autoregressive (CAR) models capture spatial correlation [16]. These methods have been validated and widely applied across various safety analysis themes and improvement strategies. However, the safety influencing factors of Chinese mountain freeways show obvious seasonal clustering and correlation, which is the most significant difference from other highways. Specifically, meteorological conditions, pavement performance and traffic composition vary considerably between seasons and follow cyclical pattern of changes. This phenomenon is specifically reflected in the crash model in parameters with temporal variations [2,5,17]. For example, the skid resistance of road surfaces deteriorates drastically in winter and rainy days, resulting in a tendency for rollover crashes due to insufficient skid resistance of road surfaces to occur during the rainy season and winter [2]. The Annual Average Daily Traffic (AADT) on mountain freeways in China increases dramatically during holidays, which exacerbates its impacts on crashes during the “Spring Festival” in winter and summer vacation, while the safety effects of the traffic volume is weakened during the season when there are fewer holidays [5]. The researches [18–22] pointed out that ignoring the temporal correlations could lead to an underestimation of parameter variance and even misidentification of influencing factors. Therefore, it is crucial to provide constructive guidance for implementing accurate crash mitigation measures by focusing on time-correlated crash modeling methods for the significant seasonal cyclical changes in risk factors unique to mountain freeways.

## 2. Literature review

### 2.1. Crash modeling methods related to temporal correlations

Time-correlation based crash modeling methods have made some positive attempts in past studies [23–25]. These methods can be categorized into two groups. The first includes generalized estimating equations (GEE) and their improved variants, which model different temporal structures, such as independent, exchangeable, autoregressive, or unstructured time terms, to capture the temporal effects of crash influences [26–28]. The second category incorporates autoregressive structural equations based on traditional counting models to describe the correlations between time-series observations and their lagged values. Notable models in this group include Bayesian hierarchical Poisson models with lag-1 autoregressive (AR-1) components [29-31], integer-valued autoregressive Poisson models [33–35], and autoregressive integrated moving average models [32]. For example, Wang et al. (2024) [27] examined the temporal correlation of three years of crash data at signal-controlled intersections using four enhanced GEE models. Their findings indicated that models incorporating autoregressive structures provided superior goodness-of-fit compared to independent, exchangeable, and unstructured time models. However, the study also highlighted that the linear correlation assumption in GEEs did not align with the complex nonlinear, time-varying patterns of crash data, which negatively impacted the model’s estimation results. Shaheed et al. (2016) [31] empirically evaluated the temporal correlation of the Iowa Department of Transportation (DOT) crash database by constructing a first-order autoregressive Bayesian Poisson model. Their results demonstrated that the first-order autoregressive effect significantly improved the representation of the temporal correlation in crash data. Sawtelle (2023) [36] investigated the effects of weather and time on crash types by analyzing annual trends and random parameter variations. Using a fully Bayesian framework, they developed four multivariate Poisson lognormal models, incorporating linear and nonlinear time trends, annual varying intercepts, and annual varying coefficients. The results confirmed that the model with varying coefficients provided the best fit.

### 2.2. Cause analysis of freeway crashes

Previous studies have explored the safety effects of four categories of factors: design features, traffic conditions, pavement performance, and weather conditions. Regarding road design features, studies [5,37] have shown that curvature, slope, and latitude are positively correlated with crash frequency; that is, higher values of these indicators are associated with an increased crash risk on road segments. There is some controversy regarding the traffic safety effects of cross-sectional indicators. Yu et al., (2014) [38] found that adding lanes would help reduce single-vehicle and multi-vehicle crashes, while Hou et al. (2018) [5] concluded that three-lane and four-lane roads increase the likelihood of lane changes and overtaking compared to two-lane roads, thereby raising the risk of crashes. In terms of traffic conditions, Zeng et al. (2023) [16] and Wen et al. (2023) [26] found that freeway segments with higher ratios of conventional cars and heavy vehicles have less stable traffic systems and thus higher crash risk, with the reason that the presence of heavy vehicles and conventional cars reduces the responsiveness of traffic flow, making it more difficult for drivers to respond to changes in the environment. In recent years, with the progress of detection methods, pavement performance indicators have been proved to have significant safety effects. Flask et al. (2013) [39] and Alnawmasi et al. (2024) [8] shown that vehicle subvert crashes are likely to occur when driving in segments with deep ruts. In addition, the interactions between the friction coefficient of pavement and rains significantly affected the traffic safety of freeways, that is, increasing the road friction coefficient significantly reduces the occurrence of crashes in rainy days [40]. In recent years, research on the safety effects of weather-related indicators has gradually become one of the main streams, and these studies have provided some unique insights: wind speed, precipitation, and visibility all have negative impacts on freeway crash frequency [16,26], while heavy rainstorms have the most significant negative impacts, and are one of the main focuses for proposing targeted improvement measures [8,41].

### 2.3. Summary of research and work direction

The research on crash modeling methods related to temporal correlations had two main shortcomings. First, for the analysis of crash risk on mountainous freeways, the advanced temporal correlation models have not been adequately validated or compared for their superiority. Second, various modeling approaches for temporal correlations can be integrated and combined, providing new perspectives for innovative modeling methods. Furthermore, traffic safety analysis of Chinese mountain freeways is partly based on practitioners’ long-term working experience, and partly based on the results of qualitative safety evaluation methods of inference. Only a few are summarized based on the estimation results of a series of traffic safety econometric models. There is still scope for improvement in the traffic safety analysis methods for the mountainous freeways in China.

**Fig1:**
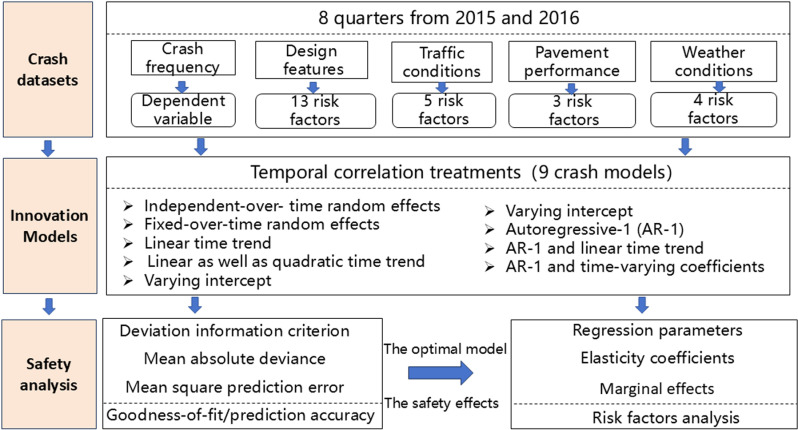
Pipeline of the proposed method.

In order to address the urgent need for safety analysis research on mountainous freeways mentioned above, this study makes three important contributions (shown in Fig. 1). Firstly, a multidimensional crash dataset involving design features, traffic conditions, pavement performance and weather conditions was established based on an eight-quarter dataset of Chinese mountainous freeways. Secondly, two new crash modelling approaches considering temporal correlation are proposed. The first model embedded an autoregressive structure and a time linear trend function within a Poisson model, while the second model incorporated an autoregressive structure and time-varying regression coefficients within a Poisson model. In addition, based on the parameter estimation results of the advanced model, an in-depth quantitative analysis of the causes of crash frequency on mountainous freeways in China is presented.

## 3. Date preparation

### 3.1. Research objects

**Fig 2:**
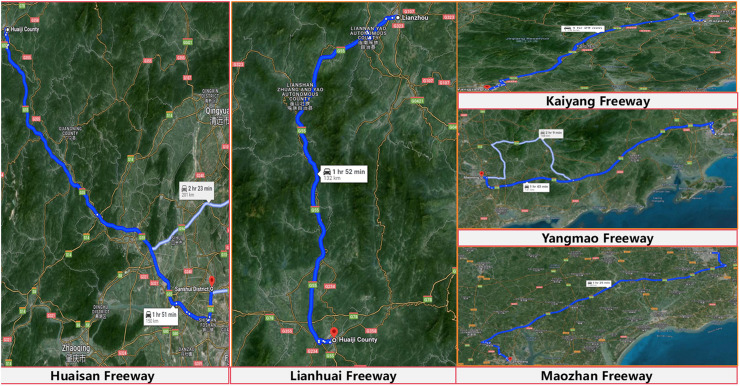
Schematic diagram of the freeway route.

The database utilized in this study encompasses five representative mountainous freeways in Guangdong Province, China. These include the G15 Expressway from Kaiping to Yangjiang (referred to as Kaiping-Yangjiang Expressway), the G15 Expressway from Yangjiang to Maoming (referred to as Yangjiang-Maoming Expressway), the G15 Expressway from Maoming to Zhanjiang (referred to as Maoming-Zhanjiang Expressway), the G55 Expressway from Lianzhou to Huaiji (referred to as Lianzhou-Huaiji Expressway), and the G55 Expressway from Huaiji to Sanshui (referred to as Huaiji-Sanshui Expressway). The dataset was collected along the routes from Kaiping to Zhanjiang and from Lianzhou to Sanshui. The route diagram and fundamental details of each highway are shown in Fig. 2, respectively.

### 3.2. Sample division methods

Due to the significant variability of traffic, pavement, and environment on mountainous freeways in China between different seasons [42–43], this paper collects and models crash data on a quarterly basis. Based on the climatic conditions in southern China, February to April, May to July, August to October, and November to January of the following year are defined as the 1st to 4th quarters, respectively. The time frame of this study is the 8 quarters in 2015-2016.

**Fig 3:**
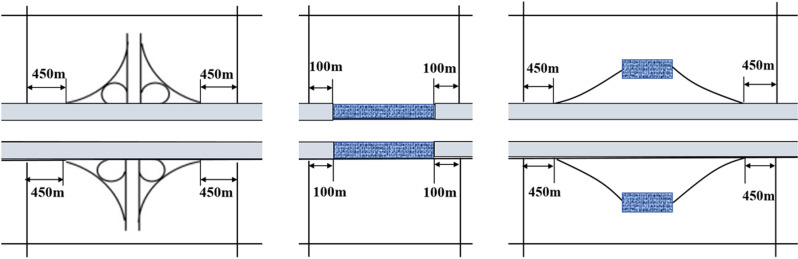
Segments type definition and division. (A) Interchange segments. (B) Tunnel segments. (C) Service-area segments.

According to the principle of indicator consistency [37], this paper cuts the freeway into segments with consistent risk factor indicators but different lengths, which circumvents the secondary processing of the original data. Specifically, truncation was carried out at the location where the road section type, plane geometry, slope and number of lanes change according to the following principles: (1) Provide that locations where the change in longitudinal slope gradient is greater than 1% are slope change points and need to be truncated here. (2) The road section types include basic sections, interchanges, service areas, and tunnel sections, and the spatial extent of each section is shown in Fig. 3. (3) Planar geometry can only be divided into straight and curved segments, while smooth and circular curves are collectively referred to as curved segments.

A total of 5568 crash modelling samples (i.e., 696 * 8) were obtained by matching 696 road sections to 8 quarters.

### 3.3. Description of variables

In this paper, 26 risk factors are initially summarized, including traffic crashes, design features, traffic conditions, pavement performance and weather conditions. Each of these variables was matched to the established sample to form a complete crash dataset (descriptive and statistical characteristics are shown in Table 1).The variables used for the model developments are extracted through the following task pipeline:

**Table 1:** Statistical characteristics of variables.

Abbr.	Description	Continuous Variables			Discrete variables	
		Mean	SD	Min/Max	Counts	Percentage (%)
Dependent variable						
NC	Number of crashes	0.746	1.383		–	–
Freeway design features						
SL	Segment length	0.877	0.31	0.172/2.691	–	–
ST	Segment types					
	0, Basic segments	–	–	–	4048	0.727
	1, interchange, tunnel or service segments.	–	–	–	1520	0.273
MBW	Middle band width					
	0, 1.5m	–	–	–	532	0.096
	1, 2m or 3m	–	–	–	5036	0.904
TER	Truck escape ramp					
	0, Do not including truck escape ramp	–	–	–	5550	0.997
	1, Including truck escape ramp	–	–	–	18	0.003
CL	Climbing lane					
	0, Do not including climbing lane	–	–	–	5428	0.975
	1,ncluding climbing lane	–	–	–	140	0.025
PT	Pavement types					
	0, cement pavement	–	–	–	5392	0.968
	1, asphalt pavement	–	–	–	176	0.032
SLI	Speed limit					
	0, Non-speed-limiting segment	–	–	–	3927	0.705
	1, Speed -limiting segment	–	–	–	1641	0.295
DS	Design speed					
	0,100km/h	–	–	–	2040	0.366
	1,120km/h	–	–	–	3528	0.634
CUR	Curvature (1/km)	0.232	0.276	0/1	–	–
CUL	Curve length (km)	1.851	1.182	0.134/3.091	–	–
SLO	Slope (%)	0.866	2.073	-4/3.968	–	–
SLL	Slope length (km)	0.922	2.088	0/4.118	–	–
PRB	Proportion of bridge	0.32	7.603	0/1	–	–
Traffic conditions						
In QADT	Quarterly average daily traffic volume (veh/day)	9.421	1.21	8.786/9.849	–	–
IVE	Quarterly percentage of Class 1 vehicles	0.741	0.042	0.630/0.836	–	–
IIVE	Quarterly percentage of Class 2 vehicles	0.027	0.015	0.008/0.050	–	–
IVVE	Quarterly percentage of Class 4 vehicles	0.024	0.014	0.005/0.053	–	–
VVE	Quarterly percentage of Class 5 vehicles	0.12	0.047	0.051/0.227	–	–
Pavement performance						
PCI	Pavement condition index	96.798	4.312	62.442/100	–	–
RQI	Riding Quality Index	94.014	2.946	65.919/99.383	–	–
SRI	Skidding Resistance Index	91.801	4.534	63.75/99.1	–	–
Weather conditions						
SMR	Quarterly percentage of days with light/medium rain	0.421	0.149	0.135/0.663	–	–
TR	Quarterly percentage of days with Torrential rain	0.066	0.057	0/0.187	–	–
WD	Quarterly percentage of days with no sustained wind	0.846	0.095	0.67/0.978	–	–
WP	Quarterly percentage of days with wind power > 4	0.173	0.2	0.090/0.890	–	–

(1) The traffic crashes were obtained from the dataset maintained in each freeway management center, which detail the locations, causes, related vehicle information, severity of the crashes and casualties. A total of 4155 crashes were recorded.
(2) Road design indicators were mainly obtained through data collection and field investigation, including the construction design drawings and record documents provided by freeway management companies. Regarding the field research, the information of geometric features and traffic facilities were obtained by manual observation and validated with driving recorders.
(3) Referring to the basis provided by Hou et al. (2018) [5], this study calculates the quarterly average daily traffic (QADT) and the percentage of each traffic composition on the basis of road segments between adjacent toll stations. Referring to the study [12], we set the segment length and QADT as exposure variables and their logarithmic values as initial inputs to the models.

(4) China’s networked freeway toll collection system categorizes vehicles into five types based on their number of axles, wheels, wheelbase, and height (as shown in Table 2). In this study, the quarterly percentages of the three types of vehicles were excluded, considering their covariance with other variables. The formulas for calculating the quarterly average daily traffic (QADT) and the weighted proportion of each type of vehicle are shown in Equations (1)–(5).

**Table 2.** Vehicle classification standard.

Vehicle types	Classification standard				Typical vehicles
	wheel base	Height of head/m	number of axles	Number of tires	
5	≥3.2	≥1.3	>3	>10	40 foot container vehicles, Heavy trailers, Heavy trucks
4	≥3.2	≥1.3	3	6-10	20 foot container vehicles, Large trucks, Large trailers, Large luxury buses
3	≥3.2	≥1.3	2	6	Large ordinary buses, Medium trucks, Medium buses,
2	≥3.2	≥1.3	2	4	Minibus, Light trucks, Minivans,
1	<3.2	<1.3	2	2-4	Pickup trucks, Cars, Motorcycles,


$$QADT_{k,t}=\frac{V_{k,1,t}+1.5V_{k,2,t}+2V_{k,3,t}+3V_{k,4,t}+3.5V_{k,5,t}}{Q_{t}}$$ (1)

$$VP_{k,1,t}=\frac{V_{k,1,t}}{V_{k,1,t}+1.5V_{k,2,t}+2V_{k,3,t}+3V_{k,4,t}+3.5V_{k,5,t}}$$ (2)

$$VP_{k,2,t}=\frac{V_{k,2,t}}{V_{k,1,t}+1.5V_{k,2,t}+2V_{k,3,t}+3V_{k,4,t}+3.5V_{k,5,t}}$$ (3)

$$VP_{k,4,t}=\frac{V_{k,4,t}}{V_{k,1,t}+1.5V_{k,2,t}+2V_{k,3,t}+3V_{k,4,t}+3.5V_{k,5,t}}$$ (4)

$$VP_{k,5,t}=\frac{V_{k,5,t}}{V_{k,1,t}+1.5V_{k,2,t}+2V_{k,3,t}+3V_{k,4,t}+3.5V_{k,5,t}}$$ (5)

Where $V_{k,1,t}$-$V_{k,5,t}$ represent the number of category 1-5 vehicles passing through segment *k* in quarter *t*. $Q_{t}$ represents the number of days in the quarter *t*. $QADT_{k,t}$ denotes the average daily traffic volume of segment *k* in quarter *t*. $VP_{k,1,t}$, $VP_{k,2,t}$, $VP_{k,4,t}$and $VP_{k,5,t}$ represent the proportions of class 1, 2, 4 and 5 vehicles in segment k and quarter t, respectively.

(5) The spatial and temporal collection frequencies of pavement performance indicators were 20/50m and 3-9 months, respectively. Therefore, the pavement performance metrics were matched to the samples by interpolation and averaging processes [2].
(6) According to the Standard for Evaluation of Highway Technical Condition (JTG H20-2007) [45], the pavement condition index (PCI) characterizes the degree of pavement damage, and a higher PCI represents a smaller degree of pavement damage. Ride Quality Index (RQI) characterizes the smoothness of the pavement, and the higher its value, the better the smoothness of the pavement. Skidding Resistance Index (SRI) is converted from the lateral force coefficient of the pavement, where the higher its value, the better the slip resistance of the pavement.
(7) Collected meteorological data, such as annual/monthly rainfall, average/maximum/minimum temperatures, and other easily obtainable weather conditions data, are widely used in road traffic safety studies [2,26,29]. For example, the results of Wen et al. (2019) [26] showed that an increase in monthly average wind speed, monthly average daily precipitation, and monthly average visibility were detrimental to freeway traffic safety. Tang et al. (2021) [2] showed that the higher the ratio of light/moderate and heavy/stormy rainfall, the higher the crash frequency in freeway tunnels, with the most pronounced effect observed for heavy/stormy rainfall. Based on this, The weather condition variables selected in this paper include the percentage of small/moderate rain days in a quarter (SMR), the percentage of torrential rain days in a quarter (TR), the percentage of days with no sustained wind in a quarter (WD), and the per-centage of days with wind power ≥  4 in a quarter (WP). The weather data used in this study were obtained from the Guangdong Meteorological Data Center and recorded at the county (district) level, and are covered by data from 41 meteorological stations, with an average distance of 36 km between stations. The shortest and longest distances between a meteorological station and a freeway are 18 km and 34 km, respectively.

## 4. Methodology

### 4.1. Model Instructions

In this study, the Poisson model was used as the basis for a fully Bayesian hierarchical model to portray the temporal correlation characteristics in the crash dataset by embedding different temporal correlation function terms in its link function respectively [46]. Specifically, in increasing order of complexity, the hierarchical models concerned with portraying temporal correlations range from independent temporal random effects to composite random time effects with AR-1 and time-varying coefficients.

#### 4.1.1. Explanation of independent spatial and spatiotemporal interaction effects.

The independent spatial effects and spatiotemporal interaction effects of crash frequency have been validated in various scenarios, including freeways [19,47], urban roads [20], and rural roads [15]. It is widely acknowledged that spatially adjacent analytical units may share common or similar underlying characteristics, which can result in spatial correlations in crash frequencies between neighboring units. A series of analytical approaches have been employed to estimate the spatial effects observed in crash data, including generalized estimating equations (GEE) [48], multilevel models [49], extended multilevel models [49], simultaneous autoregressive models (SAR) [50], conditional autoregressive models (CAR) [50], and spatial error models (SEM) [51]. Among these methods, the CAR model is the most commonly used for describing spatial associations. Xu et al. [51] pointed out that, within a Bayesian modeling framework, the CAR model can provide more accurate parameter estimates compared to other classical spatial models.

As traffic crash analysis is not limited to a single time period or geographic space, some studies have incorporated both spatial and temporal effects into their models. Bayesian hierarchical models with different spatiotemporal interaction effects have been widely applied in these studies [20,52,53]. Research has shown that in the model framework, spatiotemporal interactions are modeled using a mixture of two components, particularly a combination of structured temporal effects and unstructured spatial effects [53], which can effectively explain potential heterogeneity that cannot be accounted for by spatial or temporal effects alone.

It is important to note that the focus of this study is to investigate the temporal correlation of crash frequencies in the unique context of mountainous highways. To minimize the influence of spatial effects and spatiotemporal interaction effects, this study adopts the classical Conditional Autoregressive (CAR) model and a mixed component of structured temporal effects and unstructured spatial effects to represent the spatial associations and spatiotemporal interaction effects of crash frequencies, respectively.

#### 4.1.2. Temporal correlation models emerging from previous studies.

**Model 1: Independent temporal random effects**

The functional expression for the Poisson model is given below:


$$y_{it}\sim Poisson\left(\theta_{it}\right)$$ (6)

where, $y_{it}$ denotes the observed number of crashes on segment *i* in quarter *t*. $\theta_{it}$ denotes the average expected number of crashes on segment *i* in quarter *t*, which is assumed to have a generalized exponential form of association with each potential risk factor:


$$In\left(\theta_{it}\right)=\beta_{0}+\overset{M}{\underset{m=1}{\sum }}\beta_{m}x_{mit}+\emptyset_{i}+\varepsilon_{it}+{£}_{it}$$ (7)

where $x_{mit}$ denotes the observed value of the $m_{th}$ risk factor for segment *i* in quarter *t* and *β* is its corresponding regression coefficient. *M* denotes the number of risk factors. $\beta_{0}$ denotes the constant term of the regression coefficient, and $\varepsilon_{it}$ is the error term for segment i in quarter *t*.

Reference Yang et al. [47], In order to avoid the interference of spatial heterogeneity in the comparison of time modeling approaches, this study introduced a separate spatial random effects term $\emptyset_{i}$ (separate from the random effects) in all candidate models. A CAR (conditional autoregressive) prior characterizes the spatial random effects, which used a spatial weight matrix to describe the spatial correlation between the risk factors [45].


$$\emptyset_{i}\sim N\left(\bar{v_{i}},\frac{1}{\tau_{i}}\right)$$ (8)

$$\bar{v_{i}}=\frac{\sum_{i\neq j}v_{j}w_{ij}}{\sum_{i\neq j}w_{ij}}$$ (9)

$$\tau_{i}=\frac{\tau_{c}}{\sum_{i\neq j}w_{ij}}$$ (10)

where, $\tau_{c}$is the precision parameter in CAR prior; $w_{ij}$ is an element in the spatial weight matrix *W*, representing the spatial weight between segmentsi and *j*; Existing research has used various spatial weight structures, including various adjacency based, distance based, and semiparametric geographic weighting models [48], to analyze the spatial correlation of road entities at different scales. At the micro level, the analysis of road crash frequency often adopts an adjacency based spatial weight structure [47]. Therefore, this research also uses this structure to study spatial correlation, which stipulates that if road sections *i* andj are adjacent, $w_{ij}$=  1, otherwise $w_{ij}$=  0.

Additionally, The spatiotemporal interaction effect term of crash frequency ${£}_{it}$ is used to explain potential heterogeneity that cannot be explained by spatial and temporal effects [20]. Assuming spatiotemporal components ${£}_{it}$ follows a normal distribution with a mean of 0.


$${£}_{it}\sim N\left(0,\frac{1}{\tau_{£}}\right)$$ (11)

Where, $\tau_{£}$ is precision.

Based on the clues provided in the literature [44], we assumed that the prior distribution of the regression coefficients $\beta_{m}$ is$Normal\left(0,10^{4}\right)$, while the error term $\varepsilon_{it}$ is assumed to be normally distributed, and associated variance $\sigma_{it}$, precision $\tau_{£}$ and $\tau_{c}$ are assumed to be $Gamma\left(0.001,0.001\right)$.


$$\varepsilon_{it}∼Normal\left(0,\sigma_{it}^{2}\right)$$ (12)

$$\sigma_{it}∼Gamma\left(0.001,0.001\right)$$

$$\tau_{£}∼Gamma\left(0.001,0.001\right),\tau_{c}∼Gamma\left(0.001,0.001\right),$$ (13)

**Model 2: Time-varying fixed random effects**

The Model 1 assumed that the error term $\varepsilon_{it}$ can vary over road segments and quarters. The potential implication of this constraint is that the risk factors are assumed to be entirely independent of each road segment and time period, with no interference from spatial or temporal factors in their impact on crash occurrences. This strong assumption warrants a thorough examination to assess its alignment with real-world conditions. In contrast, Model 2 relaxes this assumption by allowing for the same unobserved characteristics of a given road segment to remain consistent across different quarters. Therefore, an identical site-specific random effect is added to the quarters in the following form:


$$In\left(\theta_{it}\right)=\beta_{0}+\overset{M}{\underset{m=1}{\sum }}\beta_{m}x_{mit}+\emptyset_{i}+\varepsilon_{i}+{£}_{it}$$ (14)

$$\varepsilon_{i}∼Normal\left(0,\sigma_{t}^{2}\right)$$ (15)

**Model 3: Linear time trend**

Based on the clues provided by the literature [30], risk factors with temporal effects may have a tendency to change approximately linearly over quarters. Therefore, a layer of linear time function structure was added to represent the pattern of change of certain risk factors over time by using time as a covariate.


$$In\left(\theta_{it}\right)=\beta_{0}+\overset{M}{\underset{m=1}{\sum }}\beta_{m}x_{m}+\beta_{m+1}t+\emptyset_{i}+\varepsilon_{i}+{£}_{it}$$ (16)

Where $\beta_{m+1}$ is a characterization parameter for the quarterly linear variation whose prior distribution is assumed to be $Normal\left(0,10^{4}\right)$.

**Model 4: Combined linear and quadratic random effects**

One of the shortcomings of Model 3 is its inability to account for the non-linear portion of the seasonal variation in time-series risk factors, which affects the results of the model’s parameter estimates. In addition to exploring linear time trends, this paper explores more complex quadratic time random effects terms [30]:


$$In\left(\theta_{it}\right)=\beta_{0}+\overset{M}{\underset{m=1}{\sum }}\beta_{m}x_{m}+\beta_{m+1}t+\beta_{m+2}t^{2}+\emptyset_{i}+\varepsilon_{i}+{£}_{it}$$ (17)

where $\beta_{m+2}$ is a parameter characterizing the trend of the quadratic quarter, which is also assumed to follow the distribution $Normal\left(0,10^{4}\right)$.

**Model 5: Intercepts-over-time random effects**

To more accurately represent the temporal trends in crash data, the model can also embed an intercept term on the temporal correlation that varies with the quarter of each segment.


$$In\left(\theta_{it}\right)=\beta_{0t}+\beta_{m}x_{m}+\emptyset_{i}+\varepsilon_{i}+{£}_{it}$$ (18)

where $\beta_{0t}$ is a vector of intercepts that vary with quarter, and the intercepts for each quarter are assumed to obey an uninformative normal prior $Normal\left(0,10^{4}\right)$.

**Model 6: coefficients-over-time random effects**

Literature [12] suggested that randomizing the regression coefficients more effectively captures spatiotemporal heterogeneity in crash frequency modeling compared to the approach of adding stratified time terms and randomizing intercepts. Consequently, we incorporated intercept coefficients and covariates that vary across different quarters.


$$In\left(\theta_{it}\right)=\beta_{0t}+\beta_{mt}x_{mt}+\emptyset_{i}+\varepsilon_{i}+{£}_{it}$$ (19)

Where, $\beta_{mt}$ is a vector of random coefficients characterizing time-varying, and each element in this vector is assumed to obey a noninformative normal prior distribution $Normal\left(0,10^{4}\right)$.

**Model 7: Autoregressive-1 (AR-1)**

Following the reference provided in the literature [30], the temporal correlations were characterized by autoregressive effects. Specifically, the distribution of $\varepsilon_{i}$ is specified as a lag-1 dependence of the error, where lag-1 indicates that time varies by quarters. AR-1 is based on the assumption of a static limit, which contains a weighted sum of the previous quarter’s values and a random term.


$$In\left(\theta_{it}\right)=\beta_{0t}+\beta_{mt}x_{mt}+\emptyset_{i}+\varepsilon_{it}+{£}_{it}$$ (20)

The weighted sum is fixed and the random term changes according to the same distribution at each time step, which means that the model is homogeneous. The distribution is as follows:


$$\varepsilon_{i1}\sim Normal\left(\frac{\sigma_{i1}^{2}}{1-\gamma^{2}}\right)$$ (21)

$$\varepsilon_{it}\sim Normal\left(\gamma \varepsilon_{it-1},\sigma_{it}^{2}\right)$$

fort > 1 (22)

whereγ is the autocorrelation coefficient ranging from -1<*γ*<1 [29]. If the autocorrelation coefficient *γ* is close to 0, then the stochastic process looks like white noise, but when |*γ*| is close to 1, the correlation between neighboring quarters is more significant. The coefficient *t* is taken as a uniform prior [26].

#### 4.1.3. The innovation model proposed in this study.

**Model 8: Combination of AR-1 and linear time trend**

The autoregressive model (Model 7) employes a lag-1 dependence of the error, taking into account the previous quarter’s error. Linear Trend (Model 3), on the other hand, used a different temporal treatment, incorporating quarterly time intervals as an explanatory variable, which may be a viable and important factor in the occurrence of crashes. Wen et al. [26] pointed out that 1st order autoregressive models are suitable for time-series variables with complex interactions, especially for traffic conditions with quarter and month as time units. While using time as an explanatory variable (linear trend model) is more concerned with exploring the changing patterns of risk factors and their impacts on crashes from the time cycle. Zeng et al. [16] verified that the time linear trend model can better portray the impacts of risk factors with significant periodicity on safety, such as traffic conditions. Therefore, this paper combines the two models’ treatments of time correlation and innovatively develops Model 8, which takes the following model form:


$$In\left(\theta_{it}\right)=\beta_{0t}+\beta_{m}x_{m}+\beta_{m+1}t+\emptyset_{i}+\varepsilon_{it}+{£}_{it}$$ (23)

where the prior distributions of the above variables all follow the same specifications as in the previous 7 models.

It is important to pointed out that temporal correlation characteristics can be specified in crash models in terms of randomization of explanatory variables and regression coefficients. A series of literatures [5,17,54] verified that the treatment of parameter randomization was more advantageous in portraying heterogeneity as opposed to explanatory variable randomization. The combination of time-varying coefficients and AR-1 can both quantify the lag-dependent correlation of adjacent time units and portray the heterogeneity of the impacts of risk factors on crashes in different time units, which is a potentially suitable modeling method for crash temporal correlations. Consequently, to investigate the effectiveness of various time-treatment combinations further, the authors introduce a new model, referred to as Model 9, which combines AR-1 and time-varying coefficients. It is expected that the standard trend of the proposed model will improve the reliability of the combined model’s performance in contrast to the conventional model. The structure of model 9 can be expressed as


$$In\left(\theta_{it}\right)=\beta_{0t}+\beta_{mt}x_{mt}+\beta_{m+1}t+\emptyset_{i}+\varepsilon_{it}+{£}_{it}$$ (24)

The prior distributions for all the aforementioned variables adhere to the same specifications as those used in the previous 8 models.

### 4.2. Model estimation and evaluation methods

Based on the clues provided in the literature [26], the candidate models were used for Bayesian parameter estimation using WinBUGS software. The candidate models were all set up with a chain of 1000,000 iterations, using the initial 70,000 iterations as an annealing treatment, and the convergence of the models was assessed by the Gelman-Rubin statistic.

Following the clue provided by reference [5], Deviation Information Criterion (DIC) is used as an assessment of goodness-of-fit, while Mean Absolute Deviation (MAD) and Root Mean Square Error (RMSE) together are used as an assessment of prediction accuracy. Specifically, DIC assesses the goodness-of-fit by combining the complexity penalty term and the bias. The MAD denotes the absolute value of the difference between the actual observations and the posterior mean, while the RMSE denotes the square root of the difference between the actual observations and the posterior mean. The specific calculation method of each performance test index is as follows:


$$DIC=\bar{D}+Pd$$ (25)

$$MAD=\frac{1}{IXT}\overset{I}{\underset{i=1}{\sum }}\overset{T}{\underset{t=1}{\sum }}\left|\bar{y_{i,t}}-y_{i,t}\right|$$ (26)

$$RMSE=\sqrt{\overset{I}{\underset{i=1}{\sum }}\overset{T}{\underset{t=1}{\sum }}{\left(\bar{y_{i,t}}-y_{i,t}\right)}^{2}/\text{N}}$$ (27)

where $\bar{D}$ represents the a posteriori mean deviation of the parameters, which is used to measure the fitting accuracy of the model, and Pd is the effective parameter, which is used to measure the complexity of the model. *N*, *I*, *T*, andC represent the number of samples, the number of road segments, the number of quarters, and the number of parameters included in the model, respectively. $\bar{y_{i,t}}$ denotes the predicted crash frequency for segment *i* in quarter *t*. $y_{i,t}$ denotes the actual crash frequency for segment *i* in quartert.

According to the clues provided by Tang et al. (2023) [2], Elastic Coefficients and Marginal Effects were further introduced to quantify the safety effects of significant factors. Specifically, the Elasticity Coefficient was used to describe the percentage change in crash frequency for each 1% increase in the continuous significant variables. The Marginal Effect represented the change degree of the average crash frequency per kilometer for each unit increase of the discrete significant variables. The calculation methods of safety effect quantitative indexes are as follows:


$$E_{x}=\frac{1}{NxT}\overset{N}{\underset{i=1}{\sum }}\overset{T}{\underset{t=1}{\sum }}\left(\frac{àu_{i,t}x_{i,t}}{u_{i,t}àu_{i,t}}\right)=\frac{1}{NxT}\overset{N}{\underset{i=1}{\sum }}\overset{T}{\underset{t=1}{\sum }}\left(\beta x_{i,t}\right)$$ (28)

$$M_{x}=\frac{1}{NxTxL}\overset{N}{\underset{i=1}{\sum }}\overset{T}{\underset{t=1}{\sum }}\frac{àu_{i,t}}{àx_{i,t}}=\frac{1}{NxTxL}\overset{N}{\underset{i=1}{\sum }}\overset{T}{\underset{t=1}{\sum }}\left[\beta \exp\left(\beta x_{i,t}\right)\right]$$ (29)

where, $E_{x}$ is the Elasticity Coefficient of continuous variable *x* on crash frequency, $x_{i,t}$ is the value ofx in segment *i* and time period *t*, *β* is the regression coefficient of $x_{i,t}$, $M_{x}$ is the Marginal Effect of discrete variable *y* on crash frequency, and *L* is the average length of modeling sample.

## 5. Results and discussion

### 5.1. Comparison of model performance

#### 5.1.1. Goodness-of-fits.

**Fig 4:**
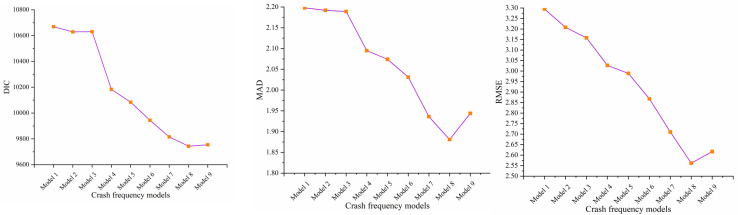
Comparison of the goodness-of-fit and prediction accuracy of the candidate models.

The Table 3 and Fig 4 showed the comparison results for goodness-of-fits. The Models 1 and 2 had the worst goodness-of-fit among all competing models, attributed to the fact that independent or fixed temporal parameters do not well characterize the temporal correlation of crash data. Adding linear or quadratic time-trend parameters improved the goodness-of-fit, but did not substantially increase the number of model parameters, as reflected in the decreasing DIC and similar $p_{d}$. for Models 1 to 4. It is worth noting that Model 6, which incorporates more time-varying coefficients than Model 5, greatly reduces the a posteriori bias, ultimately compensating for the potential complexity and resulting in a better overall fit, with a 140-point difference in DIC. Despite the fact that the AR and its variant models (Models 7 to 9) used more effective parameters, they had the best goodness-of-fit, as evidenced by their highest $p_{d}$ values. In addition, the results showed that the embedding parameter γ greatly reduced the bias ($\bar{D}$), compensating for the increase in DIC due to the inclusion of more effective parameters. Such results demonstrate the advantage of such models in flexibly adapting to influencing factors that change over the quarter, leading to a better fit to crash data. The Model 8 had the best goodness of fit of all the candidate models, with Model 9 a close second. These two new models used different approaches to produce better fits, but Model 8 used fewer effective parameters because it incorporated only linear trends as a supplement to the structural specification of the regular AR-1 model. It should be noted that the Model 9 takes advantage of the time-varying coefficients to significantly reduce the a posteriori bias (36-point difference in $\bar{D}$ values relative to Model 8), but utilizes more parameters (47-point difference in Pd values relative to Model 8) for the estimation, and therefore strikes a balance between goodness-of-fit and complexity. The inclusion of linear time-correlation parameters and time-varying coefficients significantly optimizes the number of effective parameters, which is much lower than the traditional AR-1 specification, while fully leveraging the fit provided by the AR-1 structure. Clearly, this combination controls the number of effective parameters while maintaining sufficient bias to achieve optimal goodness-of-fit, suggesting greater flexibility to adapt to crash data.

**Table 3:** Goodness-of-fit and prediction performance of alternate models.

Models	$\bar{D}$	PD	DIC	MAD	RMSE
Model 1	10248	421	10669	2.198	3.295
Model 2	10207	422	10629	2.192	3.208
Model 3	10187	443	10630	2.189	3.158
Model 4	9732	451	10183	2.095	3.027
Model 5	9580	504	10084	2.074	2.989
Model 6	9409	535	9944	2.031	2.868
Model 7	9186	629	9815	1.936	2.71
Model 8	9089	654	**9743**	**1.881**	**2.562**
Model 9	**9053**	701	9754	1.944	2.617

#### 5.1.2 . Predictive accuracy.

The predictive accuracy of Model 8 (MAD and RMSE of 1.881 and 2.562 respectively) was consistently better than other comparable models. The superiority explains the significant autocorrelation and non-linear nature of the temporal correlation of the mountain motorway crash data. Overall, the autoregressive models and its improved models (Models 7-9) outperformed the other models in all evaluation criteria, suggesting that the vector autoregressive parameters are uniquely suited for capturing the temporal correlations of crashes. The Models 1 and 2 had the worst predictive accuracies, whereas the addition of linear or quadratic trends significantly improves the predictive power (The Models 3 and 4), which showed that a portion of the temporal correlation trends in crashes can be represented by either a primary or a quadratic function structure. The sequential inclusion of intercepts or coefficients that vary over time resulted in lower MAD and RMSE values for the model. This suggested that the flexibility of the model specification contributes to the improved predictive power.

### 5.2. Estimation results of model parameters

#### 5.2.1. Hyperparameters of candidate models.

The estimation results of hyperparameters are shown in Table 4. It should be noted that the time trend parameter $\beta_{m+1}$ for the linear trend series of models (Models 3, 4, and 8) was significant at 95% Bayesian intervals of 2.142, 2.658, and 1.912, respectively, while the time trend parameter $\beta_{m+2}$ for the quadratic trend model (Model 4) was significant at 95% Bayesian intervals of 0.347. Such findings suggested that the linear and quadratic correlation function structures can capture the temporal correlation features present in crash data. Furthermore, the autocorrelation coefficients, γ, for models 7, 8, and 9 are significant of 0.417, 0.498, and 0.632 at 95% Bayesian intervals, suggesting that the autocorrelation function also portrays the temporal correlation that exists in the crash data.

**Table 4:** Estimation results of model hyperparameters.

Models	Model 1	Model 2	Model 3	Model 4	Model 5	Model 6	Model 7	Model 8	Model 9
$\beta_{0}$	-207.423	-262.589	-231.533	-239.872	-233.114	-247.224	-298.276	-243.857	-224.167
					-235.984	-246.795			-222.537
					-231.104	-249.651			-225.719
					-235.698	-247.712			-227.962
					-233.258	-246.675			-221.267
					-237.756	-251.563			-226.378
					-238.538	-245.839			-221.874
					-235.799	-248.702			-227.434
$\beta_{m+1}$			2.142	2.658				1.912	
$\beta_{m+2}$				0.347					
γ							0.417	0.498	0.632

#### 5.2.2. Regression coefficients of candidate models.

The estimation results of Regression coefficients of candidate models are shown in Table 5 and Table 6. The following conclusions can be summarized: (1) The Models 1-9 identified 11, 12, 11, 11, 12, 11, 13,13, 11 risk factors that were significant at the 95% Bayesian Confidence Interval (BCI), respectively. (2) From the sign of the regression coefficients, all significant factors had consistent safety effects in the candidate models. (3) The risk factors significantly and positively associated with crash frequency included ln(SL), ST, DS, CUR, SLO, ln(QADT), IVEV, VE, and SMR, while the risk factors significantly and negatively associated with crash frequency included CUL, PCI, RQI, SRI and TR.

**Table 5:** Estimated results of the regression coefficients (Fixed parameters).

Models	model 1		model 2		model 3		model 4		model 5		model 7		model 8	
	Mean	95%BCI	Mean	95%BCI	Mean	95%BCI	Mean	95%BCI	Mean	95%BCI	Mean	95%BCI	Mean	95%BCI
In (SL)	1.246	(0.743,2.078)	1.041	(0.556,1.522)	1.112	(1.004,1.638)	1.387	(1.021,1.977)	1.171	(0.743,1.444)	1.311	(0.773,1.643)	1.432	(0.819,1.912)
ST	0.154	(0.079,0.192)	0.124	(0.043,0.343)	0.051	(0.031,0.089)	0.126	(0.014,0.196)	0.175	(0.072,2.043)	0.176	(0.047,0.182)	0.139	(0.059,0.373)
DS	0.112	(0.087,0.154)	0.307	(0.166,0.412)			0.172	(0.051,0.219)	0.018	(0.007,0.072)	0.197	(0.059,0.231)	0.412	(0.174,0,628)
CUR	1.084	(0.796,1.465)	2.093	(1.532,2.457)	1.173	(0.921,1.652)	2.005	(1.674,2.837)	1.217	(1.352,2.442)	2.109	(1.432,3.068)	2.019	(1.281,2.771)
CUL	-0.201	(-0.274,-0.165)	-0.165	(-0.212,-0.009)	-0.322	(-0.417,-0.309,)			-0.309	(-0.594,-0.123)	-0.173	(-0.248,-0.101)	-1.074	(-1.432,-0.874)
SLO	0.013	(0.007,0.027)	0.185	(0.049,0.209)	0.281	(0.114,0.327)			0.139	(0.077,0.126)	0.146	(0.054,0.192)	0.329	(0.071,0.438)
In(QADT)	2.741	(2.053,3.121)	2.074	(1.173,3.065)	2.743	(2.012,3.452)	1.112	(1.015,1.732)	3.507	(3.143,3.828)	2.124	(1.527,2.649)	1.904	(1.097,2.712)
IVE							2.043	(0.976,3.297)			1.974	(0.928,2.239)	1.227	(0.839,1.428)
VVE	1.335	(1.016,1.643)	3.107	(2.436,4.082)	3.679	(2.195,6.875)	2.327	(1.619,2.843)	6.072	(1.753,9.048)	2.176	(1.843,2.575)	1.012	(0.631,2.084)
PCI			-0.009	(-0.022,-0.004)	-0.126	(-0.197,-0.052)	-0.013	(-0.117,-0.003)	-0.123	(-0.201,-0.053)				
RQI									-0.119	(-0.177,-0.074)	-0.006	(-0.019,-0.002)	-0.091	(-0.112,-0.044)
SRI	-0.912	(-1.412,-0.592)	-0.044	(-0.101,-0.014)	-0.033	(-0.069,-0.004)	-0.034	(-0.128,-0.002)			-0.107	(-0.132,-0.004)	-0.374	(-0.885,-0.157)
SMR	1.943	(1.391,3.112)	1.783	(1.071,2.449)	1.724	(1.040,2.341)	2.173	(1.140,2.348)	1.219	(0.574,1.843)	2.943	(1.239,3.619)	2.591	(1.326,3.448)
TR	-4.271	(-6.471,-2.916)	-6.728	(-8.112,-4.716)	-4.754	(-7.128,-3.396)	-3.298	(-4.137,-1.740)	-6.071	(-7.255,-4.982)	-5.432	(-8.771,-2.329)	-3.883	(-8.321,-5.963)

Note: (1) 95% BCI denotes 95% Bayesian Confidence Interval; (2) The table only listed risk factors that are significant at the 95% Bayesian confidence interval.

**Table 6:** Estimated results of the regression coefficients (Random parameters).

Models	model 6								model 9							
	First quarter	Second quarter	Third quarter	Fourth quarter	Fifth quarter	Sixth quarter	Seventh quarter	Eighth quarter	First quarter	Second quarter	Third quarter	Fourth quarter	Fifth quarter	Sixth quarter	Seventh quarter	Eighth quarter
In (SL)	0.943	0.960	1.309	0.816	0.881	0.987	1.240	0.677	1.074	0.749	0.743	0.940	0.853	1.116	1.114	0.811
ST	0.156	0.128	0.163	0.106	0.141	0.177	0.092	0.176	0.154	0.058	0.069	0.079	0.182	0.169	0.085	0.144
CUR	0.947	1.429	1.205	1.170	1.359	1.308	1.076	1.059	0.975	1.127	0.903	1.448	0.924	1.208	0.832	1.207
CUL	-0.230	-0.386	-0.166	-0.221	-0.225	-0.258	-0.393	-0.350								
SLO									0.138	0.205	0.106	0.166	0.107	0.201	0.190	0.132
In(QADT)	3.712	3.129	3.134	3.524	2.662	3.543	2.445	3.377	3.217	3.004	2.961	2.753	2.638	2.516	2.794	2.501
IVE	1.847	2.150	1.886	1.533	2.148	1.875	2.226	1.732	1.341	1.977	1.432	1.731	1.620	1.403	2.279	1.466
VVE	2.317	1.587	1.868	3.425	1.190	2.965	1.005	3.022	2.866	1.257	1.905	1.002	3.117	2.565	1.321	2.666
PCI	-0.090	-0.114	-0.035	-0.065	-0.044	-0.038	-0.097	-0.113	-0.077	-0.066	-0.119	-0.076	-0.079	-0.108	-0.129	-0.092
RQI	-0.086	-0.322	-0.143	-0.161	-0.226	-0.255	-0.261	-0.062								
SRI									-0.084	-0.267	-0.218	-0.239	-0.276	-0.149	-0.129	-0.094
SMR	2.629	2.331	2.122	2.332	2.924	1.834	2.022	2.231	2.796	2.658	3.094	2.505	2.473	2.183	2.469	2.571
TR	-3.867	-3.270	-3.240	-3.589	-3.383	-3.276	-3.512	-3.038	-4.008	-2.925	-3.853	-2.861	-3.860	-3.300	-3.558	-2.910

Note: (1) 95% BCI denotes 95% Bayesian Confidence Interval; (2) The table only listed risk factors that are significant at the re-95% Bayesian confidence interval.

#### 5.3. Analysis of traffic safety effects.

**Fig 5:**
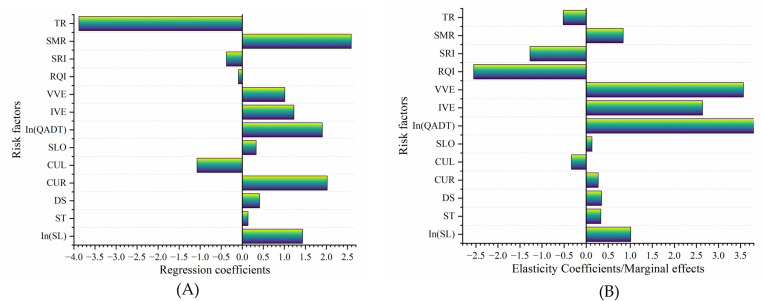
Regression coefficients and quantitative indicators of risk effects for model 8. (A) Regression coefficients of significant risk factors. (B) Elasticity coefficients/marginal effects of significant risk factors.

Combined with the Elastic Coefficients and Marginal Effect of the model 8 (as shown in Table 7 and Fig. 5), the safety effects of factors related to mountain freeway in China were deeply analyzed.

**Table 7:** Elasticity coefficients and marginal effects of risk factors in Model 8.

Variables	Elasticity Coefficients/Marginal Effects	Std. Err.	95%BCI	
			lower limit value	upper limit value
In (SL)	1.013	0.029	0.718	1.100
ST	0.334	0.026	0.411	0.392
DS	0.349	0.043	0.269	0.520
CUR	0.275	0.030	0.146	0.373
CUL	-0.333	0.078	-0.402	-0.198
SLO	0.132	0.019	0.081	0.183
In (QADT)	3.883	0.366	2.709	4.994
IVE	2.638	1.019	2.191	3.731
VVE	3.565	0.259	2.517	5.135
RQI	-2.549	1.066	-3.848	-1.980
SRI	-1.273	0.477	-2.054	-1.390
SMR	0.839	0.123	0.585	0.841
TR	-0.519	0.059	-0.544	-0.309

#### 5.3.1. Traffic safety effects of design features.

The Elasticity Coefficient of ln(SL) was 1.103, which indicated its approximately linear positive correlation with the crash frequency. The artificial segment length had no meaningful safety effects, that is, the longer the segments, the greater the natural crashes without interferences from other factors [5]. According to the Marginal Effect of ST, the crash frequency of tunnel segments, service areas and interchange segments was 0.334 times/km higher than that of basic segments on average. The presence of abundant tunnel segments on China’s mountainous freeways, whose frequent sudden environmental changes (rapid changes in vision, light, etc.) elevate the probability of nervous driving and bright and dark reactions, thus raising the crash risk [2,26]. In addition, the presence of frequent diversion and merging behaviors in the service areas and interchange segments with high traffic volumes inevitably increases the interaction behavior of vehicles thereby increasing the risk of crashes [48]. The Marginal Effect of DS showed that the segments with a Design Speed of 120km/h had an average probability of 0.349 crashes/km higher that with a Design Speed of 100km/h. In China’s mountainous freeways, the higher the design speed, the more complex the vehicle interactions were, thus increasing the probability of crashes [50]. Among the flat and longitudinal indicators, each 1% increase in the CUR and SLO indicators will increase the crash frequency by 0.275% and 0.132% respectively. A 1% increase in the CUL indicator would reduce the crash frequency by 0.333%. The sharp curved segments resulted in severely restricted sight distances and compressed the turning reflection time of high-speed vehicles, greatly reducing the tolerance of driving safety and thus increasing the risk of crashes [44]. A longer curve length means less curvature, which gives the driver sufficient time to reaction and maneuver and therefore increases the safety level [44]. To ensure the safety of the vehicle on the steep segments had a high demand for braking performance. In addition, in the traffic system with abundant heavy vehicles, the speed difference between heavy vehicles and standard vehicles intensified the instability of traffic flow in longitudinal slope segments, which is prone to traffic crashes [51].

#### 5.3.2. Traffic safety effects of traffic conditions.

The results showed that the increase of traffic volume would increase the risk of crashes. Specifically, the increase of ln(QADT) by 1% would lead to the increase of crash frequency by 3.883%. This result was verified to be applicable to freeways, urban trunk roads and rural trunk roads [5,26,37]. There were strong interactions inside the traffic system with large traffic flow, which had the negative safety effects. In terms of traffic composition, the probability of crashes was further exacerbated by the increase in IVE and VVE indicators. Specifically, a 1% increase in IVE and VVE will lead to an increase in crash frequency of 2.638% and 3.565%, respectively. Potential reasons [26] for such results were that (1) The number of Class I vehicles in this study accounts for the majority (74.1%) of the total number of vehicles, whose influences on crashes were dominant. Segments with more class 1 vehicles are bound to have more interaction effects, frequent overtaking and lane change behaviors, thus increasing the possibility of crashes. (2) The slow speeds, large volumes and slow braking responses of heavy vehicles lead to the large speed difference between vehicles, limited vision of standard cars and other unstable factors in the traffic system, which have safety risks.

#### 5.3.3. Traffic safety effects of pavement performance.

Both RQI and SRI were significantly and negatively correlated with crash frequency. Specifically, each 1% increase in RQI and SRI was associated with a 2.549% and 1.273% decrease in crash frequency, respectively. These results were consistent with the results of literatures [2,5,22,37] and were in line with the actual situation, that is, good driving comfort and skid resistance in most cases will certainly ensure effective driving safety. This reminds us that good daily road maintenance is extremely important for traffic safety. This reminds us of the important role that attention to routine road maintenance plays in improving traffic safety.

#### 5.3.4. Analysis of traffic safety effects of weather conditions.

The impacts of precipitation levels on crashes varied. SMR showed a significant positive correlation with crash frequency, while TR showed a significant negative correlation with crash frequency. Specifically, a 1% increase in SMR and TR indicators would result in a 0.839% increase and a 0.519% decrease in crash frequency, respectively. The reasons were as follows: (1) Rainy days reduced drivers’ visibility and compressed the space for safe operations. (2) The skid resistance of the road surface decreased drastically during rainy weather, seriously undermining the level of safety on mountain motorways. One possible explanation for the negative safety effects of TR is that heavy rain obstructs people’s travel plans, resulting in lower traffic volumes on freeways during heavy rains. This, in turn, reduces the probability of crashes [26,37].

### 5.4. Policy implications

The results of this study may provide some unique insights into freeway management policies.

First, road design improvements should be prioritized in high-risk areas. Features such as sharp curves and steep gradients significantly contribute to crash frequency [54,56]. Policymakers should invest in upgrading road infrastructure to incorporate gentler curves, appropriate grade transitions, and wider shoulders, particularly in crash-prone zones. Implementing advanced safety measures like guardrails, improved signage, and rumble strips can also enhance driver awareness and reduce crash risks.

Second, policy implications of high truck proportions on mountainous freeways emphasize the need for targeted interventions. Implementing dedicated truck lanes, enforcing lane-specific speed limits, and integrating dynamic weigh-in-motion systems can mitigate traffic flow disruptions and pavement damage. Additionally, enhanced training programs for truck drivers, combined with real-time monitoring and adaptive traffic regulations under adverse weather conditions, are essential to reducing crash risks and ensuring safety on mountainous freeways [55]. These measures align with sustainable road safety strategies and improve overall traffic efficiency.

Third, the findings underline the importance of regular road maintenance to ensure optimal pavement performance. Poor pavement conditions, such as cracks, potholes, or inadequate friction, are significant contributors to crashes [55, 56]. Allocating sufficient funding for timely maintenance and using advanced pavement materials and technologies to improve durability and skid resistance can substantially enhance road safety.

Weather conditions also play a critical role, particularly in mountainous areas where sudden changes in weather can lead to hazardous driving conditions [26,57]. Policymakers should focus on deploying weather-responsive traffic management systems, such as real-time weather monitoring, variable speed limit signs, and early warning systems for fog, rain, or icy conditions. Providing drivers with weather information and safety advice through digital platforms can further reduce risks.

Lastly, driver education and public awareness campaigns are crucial to improving driving behavior. Promoting defensive driving techniques, particularly for navigating challenging road conditions, can empower drivers to make safer decisions [2,37]. For professional drivers, particularly those operating freight vehicles, mandatory training on driving in mountainous terrains should be enforced.

## 6. Conclusions

The primary work of this study includes three main aspects: (1) establishing a standard safety analysis dataset that covers road design features, traffic conditions, and pavement performance; (2) proposing two novel time-correlated crash models, namely the AR-1 model combined with time-varying coefficients or linear trends, and verifying their superiority over six traditional time-correlation models; (3) systematically analyzing the quantitative impacts of various risk factors on traffic safety for mountainous freeways. The innovative model proposed in this paper is not limited to the safety analysis of mountain freeways, but can be fully extended to scenarios such as urban and rural roads, provided that the crash data have significant temporal correlations.

A practically significant contribution of this study is that the impacts of traffic composition on the safety of mountainous freeways cannot be ignored, especially the risk effects of large vehicles, which are often overlooked in safety design and operational management. Estimation of benefit-cost ratios of safety improvements for large vehicle control on mountain freeways in China is strongly recommended. If the benefit-to-cost ratio is high, practical interventions targeting the control of large vehicles should be implemented wherever possible to minimize economic losses and human casualties.

In terms of the safety effects of pavement performance, maintenance measures aimed at improving pavement smoothness, friction or reducing pavement damage can all have positive safety benefits. The deeper significance of this finding is that transportation managers can prioritize limited funds for improving pavement performance on mountain freeways while selectively spending on other aspects of roadway performance based on remaining funds.

The incorporation of accurate meteorological data has further enhanced the predictability of the safety management system, enabling management units to adopt dynamic control measures. For example, before light or moderate rain arrives, traffic control measures can be implemented in advance on special sections such as tunnels, interchanges, and service areas. Interestingly, it was found that heavy rain contributes to improving highway safety, which is contrary to common perceptions. We speculate that this is because heavy rain changes the travel plans of some road users or increases driving caution. Therefore, under financial constraints, traffic management departments should focus on safety precautions during light and moderate rain, as well as rapid response and rescue during heavy rain.

The results of this study contribute to a better understanding of crash causation on China’s mountain freeways and to the development of reliable safety countermeasures for the world’s largest mountain highway system.. The following topics warrant further study:

(1) To improve the reliability of safety analysis, modeling methods based on internal attributes such as endogeneity of crash data structures need to be studied.
(2) The safety effects of overpasses, tunnels, and service areas need to be further refined. This includes analyzing traffic volume of on-ramp and off-ramp, acceleration and deceleration lane length, interchange length, ramp speed limit, tunnel length, tunnel spacing, and other relevant factors.
(3) Although this study has collected data on crashes and risk factors from typical mountainous freeways in southern China, the issue of model transferability remains a critical challenge. Future research should aim to expand the scale of the crash dataset by incorporating data from northern China and mountainous freeways in other regions of the world with varying traffic environments, to enhance the transferability of crash prediction models.
